# The Effect of Walking Backward on a Treadmill on Balance, Speed of Walking and Cardiopulmonary Fitness for Patients with Chronic Stroke: A Pilot Study

**DOI:** 10.3390/ijerph18052376

**Published:** 2021-03-01

**Authors:** Ken-Wei Chang, Chih-Ming Lin, Chen-Wen Yen, Chia-Chi Yang, Toshiaki Tanaka, Lan-Yuen Guo

**Affiliations:** 1 Department of Sports Medicine, College of Medicine, Kaohsiung Medical University, Kaohsiung 807, Taiwan; ckw65.tw@ms.szmc.edu.tw; 2 Department of Physical Therapy, Shu-Zen Junior College of Medicine and Management, Kaohsiung 807, Taiwan; 3 Department of Neurology, Changhua Christian Hospital, Changhua 500, Taiwan; josephsimion@gmail.com; 4 Department of Mechanical and Electro-Mechanical Engineering, National Sun Yat-sen University, Kaohsiung 807, Taiwan; cmurobot@gmail.com; 5 The Master Program of Long-Term Care in Aging, College of Nursing, Kaohsiung Medical University, Kaohsiung 807, Taiwan; chiachiyang@kmu.edu.tw; 6 Center for Long-Term Care Research, Kaohsiung Medical University, Kaohsiung 807, Taiwan; 7 Department of Physical Therapy, Faculty of Health Sciences, Hokkaido University of Science, Sapporo 006-8585, Japan; tanaka-t@hus.ac.jp; 8 Ph.D. Program in Biomedical Engineering, College of Medicine, Kaohsiung Medical University, Kaohsiung 807, Taiwan; 9 Department of Medical Research, Kaohsiung Medical University Hospital, Kaohsiung 807, Taiwan

**Keywords:** chronic stroke, cardiopulmonary, Berg Balance Scale (BBS)

## Abstract

This study determines the effect of walking backward on a treadmill on balance, speed of walking and cardiopulmonary fitness for patients with chronic stroke. Subjects with chronic stroke for more than six months, whose level of Brunnstrom stage is greater than IV and who are able to walk more than eleven meters with or without assistive devices were recruited. After grouping for a single-blind clinical randomized controlled trial, the subjects were divided into two groups: eight in the control group and eight in the experimental group. All subjects were subjected to 30 min traditional physical therapy, three times a week for four weeks. The experimental group was subjected to an additional 30 min of walking backward on a treadmill. The Berg Balance Scale (BBS) and the Timed Up and Go test (TUG) were used to determine the functional balance and walking ability. The walking speed was evaluated using a timed 10-Meter Walk Test (10MWT), and the cardiopulmonary fitness was determined using a 6-Minute Walk Test (6MWT) and a pulmonary function test (PFT). All assessments were made at baseline before training commenced (pre-training) and at the end of the four-week training period (post-training). A paired *t*-test and an independent *t*-test were used to determine the effect on balance, speed of walking and cardiopulmonary fitness before and after training. The level of significance α was 0.05. After four weeks of training, the experimental group showed significant differences (*p* < 0.05) on TUG, BBS, 10MWT, 6MWT, forced vital capacity (FVC) and forced expiratory volume in one second (FEV1). This pilot study shows that the 30 min of walking backward on a treadmill three times a week for four weeks increased balance, speed of walking and cardiopulmonary fitness. Trial registration: Current Controlled Trials NCT02619110.

## 1. Introduction

A report from the World Health Organization (WHO) in 2012 shows that chronic stroke was the second highest cause of death and one of the main reasons for disability in adults [1,2]. A report from the CDC in 2008 showed that chronic stroke was the fourth highest cause of death [3]. In Taiwan, chronic stroke was the third highest cause of death. Chronic stroke occurred mostly in middle-aged and elderly people and limits movement [4], which also created problems for families [5]. Therefore, the care of stroke patients must be a patient-centered and individualized rehabilitation plan [6].

Postural control and balance were crucial for most daily activities. Some studies showed that patients with chronic stroke exhibited disturbed postural stability and poor balance because they were unable to shift their weight to hemiplegic limbs, so there was asymmetric weight-bearing and more variable endpoint trajectory [7,8,9,10]. Belgen et al. demonstrated that about 40% of patients with chronic stroke experience falls and 20% fall more than once. Patients with extensive instances of falling had poor balance and walking ability [11]. Balance and gait problems were caused by reduced postural stability, insufficient propulsion at push-off, disturbed hip and knee flexion during the swing phase and poor stability during the stance phase, so patients post-stroke had a higher risk of falling [12].

Rehabilitation is pursued to increase walking ability after chronic stroke. Walking training increased postural stability, balance, gait symmetry and walking ability in [13]. Treadmill walking was used to increase mobility for patients with chronic stroke. Previous studies showed that patients with stroke have increased walking ability and speed after walking on a treadmill [14,15,16]. However, most studies involved walking forward on the treadmill to increase balance and walking ability for patients with stroke. Few studies determined the effect of walking backward on a treadmill.

Yang et al. showed that three weeks of additional training walking backward on the ground significantly increased walking speed, stride length and gait symmetry for patients with sub-acute and chronic stroke [17]. Weng et al. showed that three weeks of additional training walking backward on a treadmill significantly increased motor function, balance and walking speed for patients with sub-acute stroke [18]. Few studies determined the effect of walking backward on a treadmill for patients with chronic stroke. This study aimed to determine the effect of walking backward on a treadmill on balance, speed of walking and cardiopulmonary fitness for patients with chronic stroke.

## 2. Materials and Methods

### 2.1. Trial Design

This study used a single-blind, randomized controlled trial. The assessor was not blinded to the group of the participants. However, all data were automatically stored on a computer and were not assessed until all participants had completed the trial. The protocol was reviewed and approved by the Institutional Review Board for Human Subject Research of Kaohsiung Medical University with ethics approval number KMUH-IRB-20130288. Prior to data collection, the purposes and procedures were fully explained, and informed consent was obtained from patients. The demographic data and clinical symptoms were obtained from interviews and medical charts. Participants were screened by the primary author using the following inclusion criteria: (1) first cerebrovascular accident history; (2) onset of stroke at least 6 months prior to enrollment in the study; (3) hemiplegia causing problems with unilateral limb movement or sensory defects; (4) Brunnstrom motor stage of lower extremity equal to or greater than IV; (5) ability to walk at least 11 m with or without assistance or ankle-foot orthosis; (6) stable medical condition to allow participation in the testing protocol and intervention; (7) no visual defects or hemianopia; (8) the ability to understand and follow the therapist’s instructions and (9) the ability to complete the training program, the pre-test, post-test and the intervention protocol. Participants were excluded if they (1) had any orthopedic problems or other conditions that affect gait (total hip replacement or total knee replacement or knee osteoarthritis); (2) had any neurological problems that produce movement disorders (Parkinson’s disease, cerebellar atrophy, epilepsy or spinal cord injury) or (3) had another uncontrolled health condition for which exercise is contraindicated (uncontrolled hypertension or a heart disease that necessitates a pacemaker, coronary heart disease, arrhythmia, rheumatic heart disease, heart failure or angina).

### 2.2. Participants

**Figure 1:**
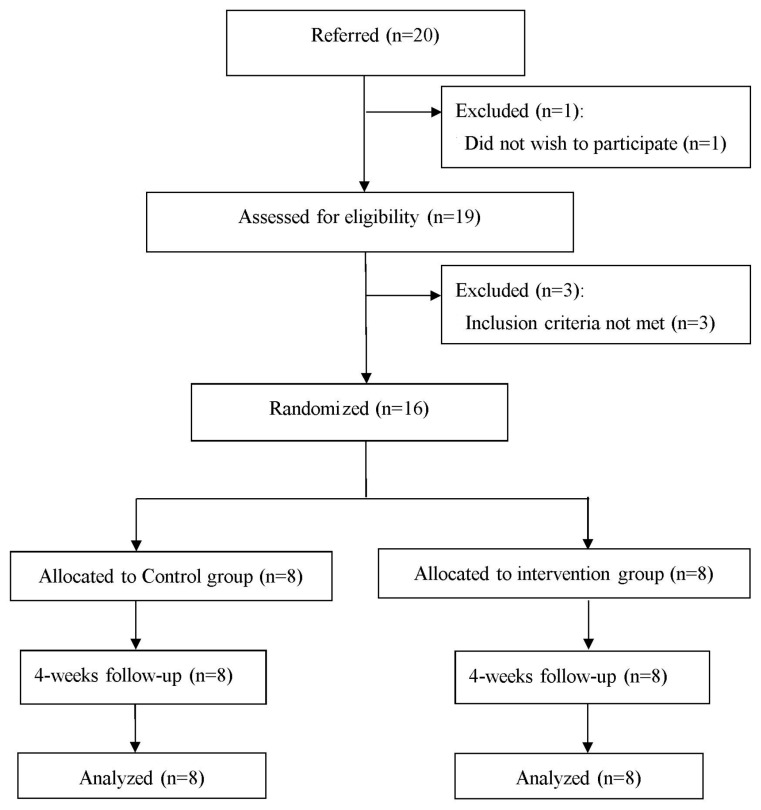
Flow diagram of the study.

Twenty chronic stroke participants were recruited from the regional teaching hospital in Kaohsiung. Prior to data collection, the purposes and procedures were fully explained and informed consent was obtained from patients. Nineteen subjects agreed to participate in the experiment and one subject did not. Three were excluded because they failed to meet the inclusion criteria, so sixteen patients with chronic stroke participated in the study and signed an informed consent document. All participants were randomly assigned to the control group or the experimental group by an independent person who picked one of the sealed envelopes before the start of the intervention. The 8 subjects in the control group underwent a conventional stroke physical therapy program and the 8 subjects in the experimental group had the conventional program supplemented with a regime of walking backward on a treadmill. All participants completed the pre- and post-test evaluation (Figure 1). Before the experiment, demographic characteristics such as stroke history, sex, age, diagnosis, stroke type (infarction or hemorrhage type), side of hemiplegia (left or right), height, weight, time of onset and duration of stroke were collected. The Brunnstrom motor stage was used to assess motion in the upper and lower extremities. The Modified Ashworth Scale (MAS) was used to assess the degree of muscular tension in the unilateral upper and lower limbs with hemiplegia [19]. The Functional Ambulation Category (FAC) was used to assess the ability to walk unaided [20].

### 2.3. Randomization and Interventions

Sixteen participants randomly drew balls from a black bag and were randomly assigned to two groups. The control group received four weeks of conventional physical therapy (3 times/week, 30 min each time). The intervention group received four weeks of conventional physical therapy and additional training walking backward on a treadmill (3 times/week, 30 min each time). The conventional physical therapy training increased strength, postural control functional mobility, and improved forward gait, but did not involve training walking backward. All conventional training and sessions that involved walking backward on a treadmill were overseen by a qualified and experienced physical therapist, who ensured that the procedure was safe and provided gait correction to decrease potential injuries and abnormal movement.

This study used a treadmill (LW1000, Dyaco, Taiwan) for which the speed setting was established based on the previous motor function of participants. The minimum speed of the treadmill was 0.2 km/h. The speed was increased slowly within the bounds of comfort for the patient. The intensity of backward walking was maintained at a comfortable level. The rating of perceived exertion (RPE) was used to monitor the degree of fatigue during backward walking on the treadmill. An exercise intensity of between RPE = 0/10 (without feeling) and RPE = 3/10 (feeling moderate and comfortable) was used. If the subject’s RPE exceeded 3/10 or the subject felt tired, dizzy, headache or nausea, training was ceased immediately to allow the subject to rest. Training was resumed only after the subject had rested and the physical therapist had assessed the subject’s condition.

### 2.4. Outcome Measures

Outcome measures included the Berg Balance Scale (BBS), a Timed Up and Go test (TUG), a 10-Meter Walk Test (10MWT), a 6-Minute Walk Test (6MWT) and a pulmonary function test (PFT). The Berg Balance Scale measures dynamic functional balance for 14 functional activities and tasks. Items on the BBS are scored on a scale from 0 to 4 points, with a maximum summed score of 56. A higher score indicates better dynamic balance and postural control. The BBS is highly correlated with the Timed Up and Go (TUG) (r = −0.76) for stroke, high internal consistency (Cronbach α = 0.92–0.98), high intra-rater (ICC = 0.97) and test–retest reliability (ICC = 0.98) [19,20,21]. The minimal detectable change (MDC) for the BBS for a patient with stroke was defined as 4.66 [22].

The Timed Up and Go test (TUG) was shown to be a reliable test for quantifying functional mobility balance and the level of fear of falling [23]. The TUG scores differentiate the patients from healthy elderly individuals, and are correlated with plantar-flexor strength (r = −0.86, *p* < 0.01), gait performance (r = 0.62–0.9; *p* < 0.05) and walking endurance (r = −0.96, *p* < 0.01) in subjects with chronic stroke [24]. Participants sat in a comfortable position on a 45–65 cm high steady chair with a backrest and armrest, with both arms resting on their thighs, and then quickly stood up and moved towards and around a fixed cone 3 m away, returned, and sat again. The tester measured the time for this process. The average time for three trials was used for analysis.

The short-distance over-ground gait speed and ability was assessed using a 10-Meter Walk Test (10MWT). The 10MWT predicts the current health of the patient. When the walking speed increased by 6 m/min, the state of health, physical function and activity were said to be improved [25]. The average score for three trials was used for analysis. Participants walked at a comfortable pace on a ten meter walkway and were timed over the middle six meters [26,27]. During the test, participants were permitted to use walking devices and/or an ankle-foot orthosis.

The 6-Minute Walk Test (6MWT) was used to measure the cardiopulmonary fitness and sport endurance [28]. Participants walked at a comfortably faster pace for 6 min to determine the cardiopulmonary fitness and sport endurance [28]. During the test, the physiotherapist accompanied the subject and the rating of perceived exertion (RPE) was used to monitor the degree of fatigue for patients with chronic stroke. Subjects could use walking devices and/or an ankle-foot orthosis without external support or assistance. During the process, subjects rested if they felt uncomfortable but the timing regime continued. When they had recovered, they walked until the total duration of exercise was six minutes.

This experiment used the pulmonary function test (Cosmed, Italy) to evaluate subjects’ cardiopulmonary fitness. Subjects underwent three tests to determine the forced vital capacity (FVC) and forced expiratory volume in one second (FEV1). Before the test, the subjects practiced the procedure twice in order to familiarize themselves with the testing procedure. After each test, the subjects rested for three minutes before taking the next test.

### 2.5. Statistical Methods

Descriptive statistics were used to analyze the baseline demographic, clinical symptom and motor function data. Continuous data used a Mann–Whitney U test and non-continuous data used a chi-square test to compare differences between the control and experimental groups. The Wilcoxon signed-rank test was used to determine the effect on balance, walking speed and cardiopulmonary fitness within the group (within-subject). The change scale from pre-test to post-test was also calculated. A Mann–Whitney U test was performed to determine the differences in all variables between the control and experimental groups. SPSS 19.0 software was used for statistical analysis. A significance level of 0.05 was used.

## 3. Results

There were no statistically significant differences in the baseline demographics and clinical symptoms between the two groups (Table 1).

Table 2 presents the clinical balance, walking ability and cardiopulmonary fitness pre-test and post-test for the control and experimental groups. There were significant improvements in the experimental group in terms of the pre-test and post-test scores for the Berg Balance Scale (BBS) (*p* = 0.008), the Timed Up and Go test (TUG) (*p* = 0.008), the timed 10-Meter Walk Test (10MWT) (*p* = 0.016), the 6-Minute Walk Test (6MWT) (*p* = 0.016), forced vital capacity (FVC) (*p* = 0.023) and forced expiratory volume in one second (FEV1) (*p* = 0.016). There was a significant improvement in the control group between pre-test and post-test for the 6-Minute Walk Test (6MWT) (*p* = 0.039).

Table 3 shows the change scale from pre-test to post-test in terms of clinical balance, walking ability and cardiopulmonary fitness between the control and experimental groups. In terms of the Berg Balance Scale (BBS), the mean change scale for the experimental group was greater than the mean change scale for the control group. There was a significant difference between groups (*p* = 0.000). For the Timed Up and Go test (TUG), the mean change scale for the control group was increased and the mean change scale for the experimental group was decreased. There was a significant difference between groups (*p* = 0.007). For the timed 10-Meter Walk Test (10MWT), the mean change in speed for the control group was decreased and the mean change in speed for the experimental group was increased. There was a significant difference between groups (*p* = 0.003). For the FEV1, the mean change scale for the control group was decreased and the mean change scale for the experimental group was increased. There was a significant difference between groups (*p* = 0.028) on the FEV1. For the 6-Minute Walk Test (6MWT), the mean change distance for the control group and the experimental group were increased. For the forced vital capacity (FVC), the mean change scale for the control group was decreased and the mean change scale for the experimental group was increased. There was no significant difference between groups on the 6MWT (*p* = 0.574) or in the forced vital capacity (*p* = 0.195).

## 4. Discussion

This pilot study demonstrates that 30 min of walking backward on a treadmill three times a week for four weeks increased performance in terms of BBS, TUG, 10MWT and FEV1. For patients with chronic stroke, rehabilitation seeks to increase independent walking ability. There are many training strategies to increase walking ability for patients with chronic stroke, but the most direct rehabilitation method uses walking training. Forward walking on a treadmill training is considered to improve posture control, balance and speed of walking [14,15,16,29,30].

Fewer studies of patients with chronic stroke involve walking backward on a treadmill. Some studies show that backward walking (BW) produces a simple temporal reversal of the kinematic and muscle activation pattern to forward walking (FW). The joint angle patterns for the time-based BW are reversed and the moment patterns for BW are similar to those for FW, except for the knee [31,32,33]. Other studies also show that the mean electromyographic (EMG) activity over the gait cycle is higher for BW than for FW because more energy is required for BW. BW achieves isometric and concentric muscle action for the vastus medialis oblique (VMO) and vastus lateralis (VL), and strengthens the knee extensor muscle [34,35], reduces patella-femoral joint compressive forces [36] and increases functional balance ability [37,38] and cardiopulmonary fitness [39,40]. However, the results of this randomized trial show that walking backward on a treadmill improved balance, speed of walking and cardiopulmonary fitness for patients with chronic stroke.

The Berg Balance Scale (BBS) is used to evaluate dynamic balance for patients with chronic stroke. The scale was used to predict the risk of falling for subjects. The mean score on the BBS for the experimental group increased by six points, which represents a statistically significant improvement. This study shows that patients with chronic stroke demonstrated increased functional balance after a regime of walking backward on a treadmill. This result is similar to those obtained in the study by Weng et al., which demonstrated that 3 weeks of additional walking backward on a treadmill significantly improved balance for patients with sub-acute stroke (average 60 days) (*p* = 0.001) [18]. These two studies included participants being in different phases post-stroke, at different times and at different intensities of training.

Hiengkaew et al. defined the minimal detectable change (MDC) for the BBS for persons with chronic stroke as a 4.66-point difference [22]. For the present study, the mean scales for the experimental group showed a 6-point difference, which exceeds the 4.66-point difference obtained by Hiengkaew et al. This study shows that dynamic balance for patients with chronic stroke is improved if a regime of walking backward on a treadmill is used. Flansbjer et al. showed that the minimal detectable change (MDC) in the TUG for patients with chronic stroke was a 2.9-s difference [41]. For the present study, the mean difference for the experimental group was 3.54-s, which exceeds this 2.9-s difference.

Our study shows that walking backward on a treadmill improved the TUG and walking ability for patients with chronic stroke. This is the first study using walking backward on a treadmill to evaluate the effect on TUG for patients with chronic stroke. Our results show that 4 weeks of walking backward on a treadmill improved the TUG and walking ability for patients with chronic stroke. BW requires more electromyographic (EMG) activity from the quadriceps muscle and more knee extensor strength than FW [34,35], so balance and motor control are increased.

The mean speed for the experimental group in this study increased by 18.4% after the WB intervention training. This is demonstrated by a statistically significant improvement in the 10-Meter Walk Test. Subjects in the experimental group walked faster after walking backward on a treadmill. This result is similar to that in the study by Weng [18]. Hiengkaew et al. showed that the minimal detectable change (MDC) in walking speed for patients with chronic stroke was a 7.8 m/min difference [22]. Previous studies show that an increase in walking speed of 6 m/min or more produces an increase in health status.

For this study, the mean difference for the experimental group was 8 m/min, which exceeds the 7.8 m/min difference for previous studies. Perry et al. [25] showed that velocity can be used to discriminate between three categories of walkers. A walking speed for patients with chronic stroke of less than 24 m/min constitutes a household walker level, a speed of between 24 and 48 m/min constitutes a limited community walker level and a speed of greater than 48 m/min constitutes a community walker [25]. For this study, the mean initial gait speed of 43.48 m/min for the experimental group increased to 51.47 m/min after four weeks of walking backward on a treadmill. The experimental group began the experiment as community walkers and were limited community walkers after the experiment, but the control group achieved no increase in walking speed. This study shows that walking backward on a treadmill increased walking speed and functional walking ability for patients with chronic stroke.

The mean distance for the experimental group for the 6-Minute Walk Test (6MWT) increased and there was a statistically significant increase in cardiopulmonary fitness from pre-training to post-training. Both groups showed an increase after training but the change was not statistically significant. The FEV1 score for the experimental group increased, but decreased for the control group. Both groups showed a statistically significant difference in terms of the change scales from pre-training to post-training. Therefore, in this study the FEV1 score increased for patients with chronic stroke after walking backward on a treadmill.

It remains uncertain whether walking backward on a treadmill improves pulmonary function. This study used a comfortable level of intensity for walking backward on a treadmill which was designed (RPE = 0/10–3/10) to ensure the safety and comfort of subjects, but this level of intensity may be insufficient to effect an increase in pulmonary function.

No side-effects or complaints were noted for any subjects, possibly because (1) the physiotherapist accompanied subjects during measurement and training so the subjects felt safe and remained calm, (2) the intensity level for the walking backward on a treadmill was comfortable, (3) the speed of the treadmill was set at 0.2 km/h during initial training, which is slower than the mean gait speed for patients with chronic stroke of 0.42 km/h and this treadmill was well-suited to patients with chronic stroke and (4) a condition for recruitment to this study was that the lower limbs of subjects were relatively stable. This study indicates that walking backward on a treadmill is a safe and effective approach for patients with chronic stroke.

This study has several limitations. The number of samples for this trial was small, so these results cannot be generalized to a larger population of persons with chronic stroke. Using ordinal measure may have impaired the ability to detect significant changes [42]. The muscular strength and electromyography of lower extremity muscles was not used to determine the effect of walking backward on a treadmill, so it is unclear whether the strength of the lower limb increased. There was no significant effect on cardiopulmonary fitness because of the low intensity of the training. This level of intensity produced no increase in cardiopulmonary fitness. Future studies might increase the intensity of training to determine the effect of walking backward on a treadmill on cardiopulmonary fitness for patients with chronic stroke. While walking backward on the treadmill, there was an uneven distribution of body weight on the subjects’ feet. Future studies might use a suspension system to distribute the body weight evenly on the feet. This study involved no follow-up, so we obtained no information about long-term effects. A follow-up is required to determine the long-term effects of additional walking backward on a treadmill. Subjects in the experimental group received more individual training than subjects in the control group during the 4-week period, so the effect of dose–response must be considered. Future studies should use larger subject groups and long-term follow-up is needed to determine whether this affects the result.

In terms of clinical application, the results of this study indicate that walking backward on a treadmill increases balance, walking speed and cardiopulmonary fitness for patients with chronic stroke. It is a safe and effective approach to achieve this. Physiotherapists play an important role in the process of walking backward on a treadmill in terms of reducing injury and the risk of falling during the training.

## 5. Conclusions

This pilot study demonstrated that an additional 30 min of walking backward on a treadmill three times a week for four weeks increased performance in terms of BBS, TUG, 10 MWT and FEV1. This pilot study demonstrated that backward walking on a treadmill training improved balance, speed of walking and cardiopulmonary fitness. Our findings suggest that walking backward on a treadmill is a helpful, important addition to chronic stroke rehabilitation.

## Figures and Tables

**Table 1:** Baseline demographics and clinical signs for control and experimental groups.

	Control (*n* = 8)	Experimental (*n* = 8)	*p*-Value
Age (years) ^a^	54.38 ± 14.05	52.39 ± 6.06	0.161 ^c^
BMI ^a^	24.43 ± 3.78	23.03 ± 1.87	0.721 ^c^
Months post stroke	43.64 ± 32.69	22.93 ± 13.7	0.266 ^c^
Gender (female/male) ^b^	3/5	2/6	0.590 ^d^
Type of stroke (hemorrhage/infarction) ^b^	3/5	7/1	0.039 ^d,^*
Hemiplegic side (left/right) ^b^	1/7	5/3	0.039 ^d,^*
Hypertension (no/yes) ^b^	2/6	5/3	0.131 ^d^
Diabetes mellitus (no/yes) ^b^	6/2	7/1	0.552 ^d^
Brunnstrom stage Proximal of UE (II/III/IV/V) ^b^	0/4/1/3	2/3/0/3	0.370 ^d^
Distal of UE (II/III/IV/V) ^b^	0/4/1/3	4/1/0/3	0.079 ^d^
LE (IV/V) ^b^	5/3	6/2	0.590 ^d^
MAS Elbow (0/I/I^+^/II) ^b^	1/3/2/2	2/1/0/5	0.202 ^d^
Knee (0/I/I^+^/II) ^b^	4/4/0/0	2/6/0/0	0.302 ^d^
Ankle (0/I/I^+^/II) ^b^	2/2/3/1	1/1/5/1	0.761 ^d^
FAC (IV/V) ^b^	3/5	4/4	0.614 ^d^
BBS ^a^	38.88 ± 8.44	36.38 ± 8.38	0.594 ^c^
TUG (seconds) ^a^	18.1 ± 8.42	24.82 ± 17.16	0.505 ^c^
10MWT (meters/minute) ^a^	49.79 ± 21.56	43.48 ± 31.03	0.574 ^c^
6MWT (meters) ^a^	227.50 ± 104.30	227.11 ± 142.72	1.000 ^c^
FVC (liters) ^a^	2.09 ± 0.49	2.37 ± 0.53	0.442 ^c^
FEV1 (liters) ^a^	1.93 ± 0.40	2.06 ± 0.39	0.505 ^c^

^a^: Mean ± standard deviation; ^b^: Number; ^c^: Mann–Whitney U test; ^d^: chi-square test; * *p* < 0.05; BMI: body mass index; MAS: Modified Ashworth Scale; FAC: Functional Ambulatory Category; BBS: Berg Balance Scale; TUG: Timed Up and Go test; 10MWT: 10-Meter Walk Test; 6MWT: 6-Minute Walk Test; FVC: forced vital capacity; FEV1: forced expiratory volume in one second.

**Table 2:** Pre-test and post-test results for clinical balance, walking ability and cardiopulmonary fitness within the control and experimental groups.

	Control (*n* = 8)			Experimental (*n* = 8)		
	Pre-Test	Post-Test	*p*-Value ^b^	Pre-Test	Post-Test	*p*-Value ^b^
Functional balance, BBS ^a^	38.9 ± 8.4	39.4 ± 8.4	0.25	36.4 ± 8.4	42.4 ± 9.8	0.01 *
Functional walking ability, TUG (seconds) ^a^	18.1 ± 8.4	18.5 ± 8.3	0.95	24.8 ± 17.2	21.3 ± 15.9	0.01 *
Speed of walking (comfortable) 10MWT (meters/minute) ^a^	49.8 ± 21.6	48.6 ± 20.8	0.74	43.5 ± 31.0	51.5 ± 36.6	0.02 *
Cardiopulmonary						
6MWT (meters) ^a^	227.5 ± 104.3	249.7 ± 113.8	0.04	227.1 ± 142.7	255.1 ± 166.1	0.02 *
FVC (liters) ^a^	2.1 ± 0.5	2.0 ± 0.4	0.55	2.4 ± 0.5	2.5 ± 0.6	0.02 *
FEV1 (liters) ^a^	1.9 ± 0.4	1.9 ± 0.3	0.46	2.1 ± 0.4	2.3 ± 0.5	0.02 *

^a^: Mean ± standard deviation; ^b^: Wilcoxon signed-rank test; *: *p* < 0.05.

**Table 3:** Change scale from pre-test to post-test for clinical balance, walking ability and cardiopulmonary fitness between the control and experimental groups.

	Control (*n* = 8)	Experimental (*n* = 8)	
	Post-Test–Pre-Test	Post-Test–Pre-Test	*p*-Value ^b^
Functional balance, BBS ^a^	0.50 (−0.13, 1.13)	6.00 (3.9, 8.1)	0.000 *
Functional walking ability, TUG (seconds) ^a^	0.37 (−1.89, 2.63)	−3.54 (−5.62, −1.46)	0.007 *
Speed of walking (comfortable), 10MWT (meters/minute) ^a^	−1.22 (−4.49, 2.05)	8.00 (2.96, 13.04)	0.003 *
Cardiopulmonary			
6MWT (meters) ^a^	22.18 (−3.22, 47.59)	28.02 (3.13, 52.91)	0.574
FVC (liters) ^a^	−0.08 (−0.29, 0.13)	0.11 (0.01, 0.21)	0.195
FEV1 (liters) ^a^	−0.07 (−0.25, 0.11)	0.19 (0.04, −0.33)	0.028 *

^a^: Mean (95% CI); ^b^: Mann–Whitney U test; *: *p* < 0.05.

## References

[1] Mathers C.D., Loncar D. (2006). Projections of global mortality and burden of disease from 2002 to 2030. PLoS Med..

[2] Mendis S. (2013). Stroke disability and rehabilitation of stroke: World Health Organization perspective. Int. J. Stroke.

[3] Miniño A.M., Murphy S.L., Xu J., Kochanek K.D. (2011). Deaths: Final data for 2008. Natl. Vital Stat..

[4] Bonita R., Beaglehole R. (1988). Recovery of motor function after stroke. Stroke.

[5] Chen C.Y., Huang Y.B., Tzu-Chi Lee C. (2013). Epidemiology and disease burden of ischemic stroke in Taiwan. Int. J. Neurosci..

[6] Perin C., Bolis M., Limonta M., Meroni R., Ostasiewicz K., Cornaggia C.M., Alouche S.R., da Silva Matuti G., Cerri C.G., Piscitelli D. (2020). Differences in Rehabilitation Needs after Stroke: A Similarity Analysis on the ICF Core Set for Stroke. Int. J. Environ. Res. Public Health.

[7] Niam S., Cheung W., Sullivan P.E., Kent S., Gu X. (1999). Balance and physical impairments after stroke. Arch. Phys. Med. Rehabil..

[8] Eng J.J., Chu K.S. (2002). Reliability and comparison of weight-bearing ability during standing tasks for individuals with chronic stroke. Arch. Phys. Med. Rehabil..

[9] Hu C.-Y., Tsai S.-C., Chen C.-N.J., Wang F.-T., Liao Y.-H. (2020). The rehabilitation exercise and nutrition/supplement strategy in chronic stroke survivor: Possible physiological mechanisms and practical applications. Phys. Educ. J..

[10] Tomita Y., Turpin N.A., Piscitelli D., Feldman A.G., Levin M.F. (2020). Stability of reaching during standing in stroke. J. Neurophysiol..

[11] Belgen B., Beninato M., Sullivan P.E., Narielwalla K. (2006). The association of balance capacity and falls self-efficacy with history of falling in community-dwelling people with chronic stroke. Arch. Phys. Med. Rehabil..

[12] Aizen E. (2014). Falls in patients with stroke. Harefuah.

[13] Liao Y., Lin C.-Y., Park H., Oka K. (2018). A systematic review of environmental factors and older adults’ walking behavior. Phys. Educ. J..

[14] Moseley A.M., Stark A., Cameron I.D., Pollock A. (2003). Treadmill training and body weight support for walking after stroke. Cochrane Database Syst. Rev..

[15] Charalambous C.C., Bonilha H.S., Kautz S.A., Gregory C.M., Bowden M.G. (2013). Rehabilitating walking speed poststroke with treadmill-based interventions: A systematic review of randomized controlled trials. Neurorehabilit. Neural Repair.

[16] Dean C.M., Ada L., Bampton J., Morris M.E., Katrak P.H., Potts S. (2010). Treadmill walking with body weight support in subacute non-ambulatory stroke improves walking capacity more than overground walking: A randomised trial. J. Physiother..

[17] Yang Y.R., Yen J.G., Wang R.Y., Yen L.L., Lieu F.K. (2005). Gait outcomes after additional backward walking training in patients with stroke: A randomized controlled trial. Clin. Rehabil..

[18] Weng C.S., Wang J., Pan X.Y., Yu Z.Z., Wang G., Gao L.P., Huo C.N. (2006). Effectiveness of backward walking treadmill training in lower extremity function after stroke. Zhonghua Yi Xue Za Zhi.

[19] Berg K.O., Maki B.E., Williams J.I., Holliday P.J., Wood-Dauphinee S.L. (1992). Clinical and laboratory measures of postural balance in an elderly population. Arch. Phys. Med. Rehabil..

[20] Berg K.O., Wood-Dauphinee S.L., Williams J.I., Maki B. (1992). Measuring balance in the elderly: Validation of an instrument. Can. J. Public Health.

[21] Berg K., Wood-Dauphinee S., Williams J.I. (1995). The Balance Scale: Reliability assessment with elderly residents and patients with an acute stroke. Scand. J. Rehabil. Med..

[22] Hiengkaew V., Jitaree K., Chaiyawat P. (2012). Minimal detectable changes of the Berg Balance Scale, Fugl-Meyer Assessment Scale, Timed “Up & Go” Test, gait speeds, and 2-minute walk test in individuals with chronic stroke with different degrees of ankle plantarflexor tone. Arch. Phys. Med. Rehabil..

[23] Podsiadlo D., Richardson S. (1991). The timed “Up & Go”: A test of basic functional mobility for frail elderly persons. J. Am. Geriatr. Soc..

[24] Ng S.S., Hui-Chan C.W. (2005). The timed up & go test: Its reliability and association with lower-limb impairments and locomotor capacities in people with chronic stroke. Arch. Phys. Med. Rehabil..

[25] Perry J., Garrett M., Gronley J.K., Mulroy S.J. (1995). Classification of walking handicap in the stroke population. Stroke.

[26] Bohannon R.W., Andrews A.W., Thomas M.W. (1996). Walking speed: Reference values and correlates for older adults. J. Orthop. Sports Phys. Ther..

[27] Bohannon R.W. (1997). Comfortable and maximum walking speed of adults aged 20–79 years: Reference values and determinants. Age Ageing.

[28] Balke B. (1963). A simple field Test for the assessment of physical fitness. rep 63-6. Rep. Civ. Aeromed Res. Inst. US.

[29] Da Cunha I.T., Lim P.A., Qureshy H., Henson H., Monga T., Protas E.J. (2002). Gait outcomes after acute stroke rehabilitation with supported treadmill ambulation training: A randomized controlled pilot study. Arch. Phys. Med. Rehabil..

[30] Ribeiro T., Britto H., Oliveira D., Silva E., Galvão E., Lindquist A. (2013). Effects of treadmill training with partial body weight support and the proprioceptive neuromuscular facilitation method on hemiparetic gait: A randomized controlled study. Eur. J. Phys. Rehabil. Med..

[31] Grasso R., Bianchi L., Lacquaniti F. (1998). Motor patterns for human gait: Backward versus forward locomotion. J. Neurophysiol..

[32] Carpenter M.G., Bellos A., Patla A.E. (1998). Is backward stepping over obstacles achieved through a simple temporal reversal of forward stepping?. Int. J. Neurosci..

[33] Winter D.A., Pluck N., Yang J.F. (1989). Backward walking: A simple reversal of forward walking?. J. Mot. Behav..

[34] Flynn T.W., Soutas-Little R.W. (1993). Mechanical power and muscle action during forward and backward running. J. Orthop. Sports Phys. Ther..

[35] Cipriani D.J., Armstrong C.W., Gaul S. (1995). Backward walking at three levels of treadmill inclination: An electromyographic and kinematic analysis. J. Orthop. Sports Phys. Ther..

[36] Flynn T.W., Soutas-Little R.W. (1995). Patellofemoral joint compressive forces in forward and backward running. J. Orthop. Sports Phys. Ther..

[37] Hao W.Y., Chen Y. (2011). Backward walking training improves balance in school-aged boys. Sports Med. Arthrosc. Rehabil. Ther. Technol..

[38] Zhang X., Zhang Y., Gao X., Wu J., Jiao X., Zhao J., Lv X. (2014). Investigating the role of backward walking therapy in alleviating plantar pressure of patients with diabetic peripheral neuropathy. Arch. Phys. Med. Rehabil..

[39] Chaloupka E.C., Kang J., Mastrangelo M.A., Donnelly M.S. (1997). Cardiorespiratory and metabolic responses during forward and backward walking. J. Orthop. Sports Phys. Ther..

[40] Myatt G., Baxter R., Dougherty R., Williams G., Halle J., Stetts D., Underwood F. (1995). The cardiopulmonary cost of backward walking at selected speeds. J. Orthop. Sports Phys. Ther..

[41] Flansbjer U.B., Holmbäck A.M., Downham D., Patten C., Lexell J. (2005). Reliability of gait performance tests in men and women with hemiparesis after stroke. J. Rehabil. Med..

[42] Gorter R., Fox J.P., Apeldoorn A., Twisk J. (2016). Measurement model choice influenced randomized controlled trial results. J. Clin. Epidemiol..

